# Expanding the *COL4A4* variant spectrum: genotype-phenotype correlation in 19 Chinese children using updated Alport kidney disease classification

**DOI:** 10.1080/0886022X.2025.2570072

**Published:** 2025-10-14

**Authors:** Yutong Huang, Hongzhou Lin, Huihui Chen, Qifan Zhu, Xinyi Jiang, Xiu-Feng Huang, Dexuan Wang

**Affiliations:** ^a^ Department of Pediatrics, The Second Affiliated Hospital and Yuying Children’s Hospital of Wenzhou Medical University, Wenzhou, Zhejiang, China; ^b^ Zhejiang Provincial Clinical Research Center for Pediatric Disease, The Second Affiliated Hospital and Yuying Children’s Hospital of Wenzhou Medical University, Wenzhou, Zhejiang, China

**Keywords:** Alport syndrome, *COL4A4*, next-generation sequencing, hematuria, pediatrics

## Abstract

**Background: **Variants in the *COL4A4* gene have been identified as a significant cause of autosomal Alport syndrome. This study aimed to investigate the genetic features, clinical manifestations, and genotype-phenotype correlations in Chinese children with *COL4A4* variants.

**Methods: **19 children with *COL4A4* variants who presented with hematuria and/or proteinuria as their main complaints were included in our analysis. Genetic variants were identified using next-generation sequencing, Sanger sequencing and comprehensive bioinformatics analysis. Clinical data and family histories were retrospectively collected.

**Results: **A total of 17 distinct *COL4A4* variants were identified, 10 of which had not been previously described (10/17). Among these novel variants, 3 were likely pathogenic variants (3/10) and 1 was pathogenic variant (1/10). Among 19 patients, two carried compound heterozygous variants, one was homozygous, and the remainder were heterozygous. Most children presented with hematuria (4 had gross hematuria and 15 microscopic) and had a family history of kidney disease, with no extrarenal manifestations. In our cohort, frameshift, and missense *COL4A4* variants were related to varying degrees of Alport kidney disease and nonsense variants resulted in isolated hematuria.

**Conclusion: **This is the first study to apply the new classification of Alport kidney disease to Chinese pediatric patients with *COL4A4* variants, substantially expanding the spectrum of *COL4A4* variants and providing new insights into genotype–phenotype correlations in this population.

## 1. Introduction

The *COL4A4* gene encodes the type IV collagen α4 chain, which features an N-terminal 7S domain, a triple-helical collagenous domain, and a C-terminal non-collagenous (NC1) domain [1]. The type IV collagen α4 chain, along with the highly homologous α3(IV) and α5(IV) chains, assembles into a heterotrimeric collagen structure that plays a critical role in the glomerular basement membrane (GBM) and its barrier function [2]. Variants in type IV collagen genes result in the loss or dysfunction of the collagen IVα345 scaffold, leading to a spectrum of diseases ranging from thin basement membrane nephropathy (TBMN) to Alport syndrome (AS, OMIM #301050) which is characterized by hematuria, proteinuria, progressive kidney dysfunction, sensorineural hearing loss, and ocular abnormalities [3,4]. In AS, renal pathology typically shows diffuse and uneven thickening of the GBM, with features such as splitting, tearing, lamellation, or a basket-weave pattern [3].

Alport syndrome is generally classified into X-linked Alport syndrome (XLAS), caused by variants in the *COL4A5* gene, and autosomal dominant (ADAS) or autosomal recessive (ARAS) forms, resulting from variants in *COL4A3* or *COL4A4* genes [5]. XLAS accounts for approximately 80% of AS cases, ARAS for about 15%, and ADAS for 5% [6]. TBMN, also known as benign familial hematuria (historical term), is an autosomal dominant condition characterized by persistent or recurrent glomerular hematuria, thinning of the GBM, and normal kidney function [7]. Heterozygous pathogenic variants in *COL4A3* or *COL4A4* are commonly observed in patients with familial hematuria, with affected individuals rarely developing hearing or ocular abnormalities [8]. TBMN generally follows a favorable clinical course without specific treatment; however, 10% to 20% of patients may progress to kidney failure [2].

The traditional nomenclature for diseases caused by type IV collagen gene variants has been regarded as outdated and confusing. As a result, Kashtan et al. [9] proposed a revised terminology for Alport syndrome that highlights heterozygous individuals who do not fulfill criteria for classic disease, thereby facilitating earlier diagnosis and intervention. This scheme was recently refined and extended by Puapatanakul & Miner [10] into a four-tier classification of collagen IVα345-associated kidney disease: severe classic AS, mild to moderate AS, proteinuric/nephrotic Alport kidney disease with focal segmental glomerulosclerosis (FSGS), and hematuric Alport kidney disease.

In this study, we adopt this updated classification and retrospectively analyze the clinical and pathological data of Chinese children with *COL4A4* variants. Our goal is to expand the variant spectrum of the *COL4A4* gene, explore genotype-phenotype correlations, and provide a more reliable foundation for disease diagnosis and prognosis.

## 2. Materials and methods

### 2.1. Patients and data collection

Nineteen children harboring isolated *COL4A4* variants (excluding any additional genetic causes of hematuria or proteinuria), admitted to The Second Affiliated Hospital and Yuying Children’s Hospital of Wenzhou Medical University due to hematuria (gross and microscopic) or proteinuria found on a routine physical examination, were enrolled in this study between January 2017 and April 2024. Written informed consent was obtained from all participants or their guardians in accordance with the principles of the Declaration of Helsinki. Ethical approval was granted by the Institutional Review Board and the Medical Ethics Committee of The Second Affiliated Hospital and Yuying Children’s Hospital of Wenzhou Medical University (2023-K-148-02). Clinical data were collected retrospectively, including sex, age of first detection of urinary abnormalities, clinical presentation, examination records, treatment history, disease progression, age and clinical features at last follow-up, and family history of kidney disease. Kidney biopsies were performed on available patients for light microscopy, electron microscopy, and immunofluorescence.

### 2.2. Genetic analysis and variant evaluation

Genomic DNA (gDNA) was extracted from EDTA-anticoagulated peripheral blood samples from participants and their family members, and whole-exome sequencing was performed using IDT’s xGen Exome Research Panel v2.0. Conservation estimation and pathogenicity prediction were performed by online bioinformatics tools, such as Mutation Taster (https://www.mutationtaster.org), SIFT (http://provean.jcvi.org/index.php), Provean (http://provean.jcvi.org/), PolyPhen-2 (http://genetics.bwh.harvard.edu/pph2/]). Additionally, SpliceAI (https://spliceailookup.broadinstitute.org/) and HSF Pro (https://hsf.genomnis.com/) were used for predicting potential splicing alterations in intronic variants and frameshift variants. The version number of the relevant reference sequence was NM_000092.5 in this study. Variants were classified according to the guidelines of the American College of Medical Genetics and Genomics (ACMG) [11]. After polymerase chain reaction (PCR), the target sequence variants were verified by Sanger sequencing in probands and available family members [12]. The variants were further analyzed in the Human Gene Mutation Database (HGMD) and the ClinVar databases (last accessed 29 March 2025).

### 2.3. Statistical analysis

Quantitative data with normal distribution were expressed as means ± standard deviations (mean ± SD), while non-normally distributed data were expressed as medians (25th-75th percentiles). Qualitative data were expressed as counts and percentages. The Mann-Whitney U test was used to compare the age of onset between the truncating and non-truncating variant groups. Pearson’s chi-squared test (χ^2^) or Fisher’s exact test was used to analyze qualitative data. A p-value of less than 0.05 was considered statistically significant.

## 3. Results

### 3.1. Molecular characterization of COL4A4 variants

**Figure 1.**
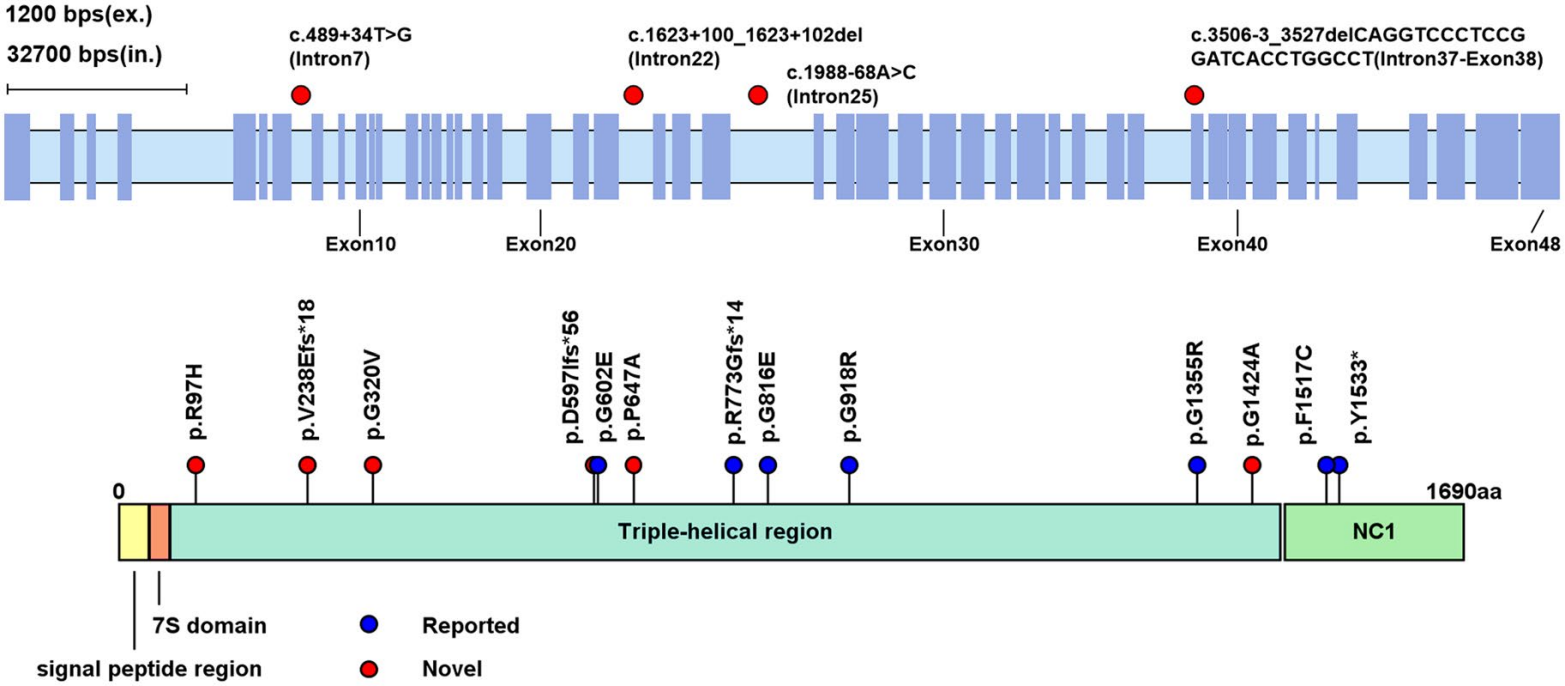
The locations of the identified 17 *COL4A4* variants.

The genetic details of the *COL4A4* variants identified in this study are summarized in Table 1 (the ACMG classification criteria in Supplementary Table 2), with their locations illustrated along the protein structure in Figure 1. A total of 72 children with hematuria or proteinuria found during physical examinations underwent whole exome sequencing and 19 children with isolated *COL4A4* variants were enrolled in our study, including one carrying homozygous variants and two carrying compound heterozygous variants. We identified 17 distinct *COL4A4* variants, consisting of 9 missense variants, 4 frameshift variants, 3 intronic variants, and 1 nonsense variant, all confirmed by Sanger sequencing. Of these, 3 were classified as pathogenic (P), 6 as likely pathogenic (LP), and 8 as variants of uncertain significance (VUS) according to the American College of Medical Genetics and Genomics (ACMG) guidelines. The most recurrent *COL4A4* variant was a previously reported pathogenic nonsense variant p.Y1533* accounting for 18.2% (4/22) of alleles, followed by a novel missense variant p.P647A (2/22, 9.1%) which was classified as VUS. Among the missense variants, glycine substitution was the most prevalent, accounting for 70.0% (7/10). Ten of the seventeen distinct variants identified were novel (last accessed 29 March 2025). Novel variants are indicated in red, and previously reported variants are in blue. The four intronic variants are mapped on the *COL4A4* gene, while the remaining exonic variants are shown on the *COL4A4* protein domain map (Figure 1).

**Table 1.** Characteristics of *COL4A4* variants identified in our study.

Pt. No.	Genetic variant (cDNA; protein)	Zygosity	Variant classification	Exon/Intron	SIFT^a^/PROVEAN^b^/ PolyPhen-2^c^/ Mutation taster^d^	SpliceAI Δscore	gnomAD	HGMD^e^	Clinvar^f^	ACMG class^f^	Reported/Novel	Clinical presentation^g^
1	c.2752G > A (p.G918R)	Het	Missense	Exon31	D/D/D/D	0	0.000012	DM	P	P	Reported	MH
2	c.2317_2318del (p.R773Gfs*14)	Het	Frameshift	Exon28	./././D	0.01	/	/	LP	LP	Reported	GH; PRO
3	c.1785del (p.D597Ifs*56)	Het	Frameshift	Exon24	./././D	0	/	/	/	LP	Novel	MH
4	c.4599T > G (p.Y1533*)	Het	Nonsense	Exon47	./././D	/	/	DM	P/LP	P	Reported	MH
5	c.290G > A (p.R97H)	Het	Missense	Exon5	T/N/B/N	0	0.000014	/	VUS	VUS	Novel	MH
6	c.4550T > G (p.F1517C)	Het	Missense	Exon47	D/D/D/D	/	/	/	VUS	VUS	Reported	MH
7	c.1939C > G (p.P647A)	Het	Missense	Exon25	D/D/D/N	0	0.000001	/	/	VUS	Novel	MH; PRO
8	c.4063G > A (p.G1355R)	Het	Missense	Exon42	D/D/D/D	/	/	/	LP/VUS	LP	Reported	GH
9	c.3506-3_3527del (p.?)	Het	Frameshift	Intron37-Exon38	./././.	0.98	/	/	/	VUS	Novel	GH
10	c.4271G > C (p.G1424A)	Het	Missense	Exon45	D/D/D/D	0	0.000001	/	/	LP	Novel	MH
11	c.4599T > G (p.Y1533*)	Het	Nonsense	Exon47	./././D	/	/	DM	P/LP	P	Reported	MH
12	c.1939C > G (p.P647A)	Het	Missense	Exon25	D/D/D/N	0	0.000001	/	/	VUS	Novel	MH; PRO
13	c.1805G > A (p.G602E)	Het	Missense	Exon25	D/D/D/D	/	/	/	VUS	LP	Reported	MH
14	c.711_726del (p.V238Efs*18)	Het	Frameshift	Exon12	./././D	0.21	/	/	/	P	Novel	MH
15	c.4599T > G (p.Y1533*)	Het	Nonsense	Exon47	./././D	/	/	DM	P/LP	P	Reported	MH
16	c.959G > T (p.G320V)	Hom	Missense	Exon16	D/D/D/D	/	/	/	/	LP	Novel	MH; PRO
17	c.4599T > G (p.Y1533*)	Het	Nonsense	Exon47	./././D	/	/	DM	P/LP	P	Reported	MH
18	c.2447G > A (p.G816E)	Compound Het	Missense	Exon29	D/D/D/D	0.01	0.000011	DM	LP/VUS	VUS	Reported	GH; PRO; edema
	c.1623 + 100_1623 + 102del (p.?)		Intron	Intron22	./././.	0.04	/	/	/	VUS	Novel	
19	c.489 + 34T > G (p.?)	Compound Het	Intron	Intron7	./././.	0.90	/	/	/	VUS	Novel	MH
	c.1988-68A > C (p.(=))		Intron	Intron25	./././.	0	/	/	/	VUS	Novel	

SIFT, Sorting Intolerant From Tolerant; PROVEAN, Protein Variation Effect Analyzer; PolyPhen-2, Polymorphism Phenotyping v2.

HGMD, Human Gene Mutation Database; ACMG, American College of Medical Genetics and Genomics. RefSeq NM_000092.5.

^a^ SIFT: D, deleterious; T, tolerated.

^b^ PROVEAN: D, Deleterious; N, Neutral.

^c^ PolyPhen-2: D, probably damaging; P, possibly damaging; B, Benign.

^d^ Mutation taster: A, disease causing automatic; D, disease causing; N, polymorphism; P, polymorphism automatic.

^e^ HGMD: DM, Disease-causing mutation.

^f^ Clinvar/ACMG class: P, Pathogenic; LP, Likely Pathogenic; VUS, Variant of uncertain significance.

^g^ Clinical presentation: MH, microscopic hematuria; GH, gross hematuria; PRO, proteinuria.

### 3.2. Clinical features of patients with COL4A4 variant

**Figure 2.**
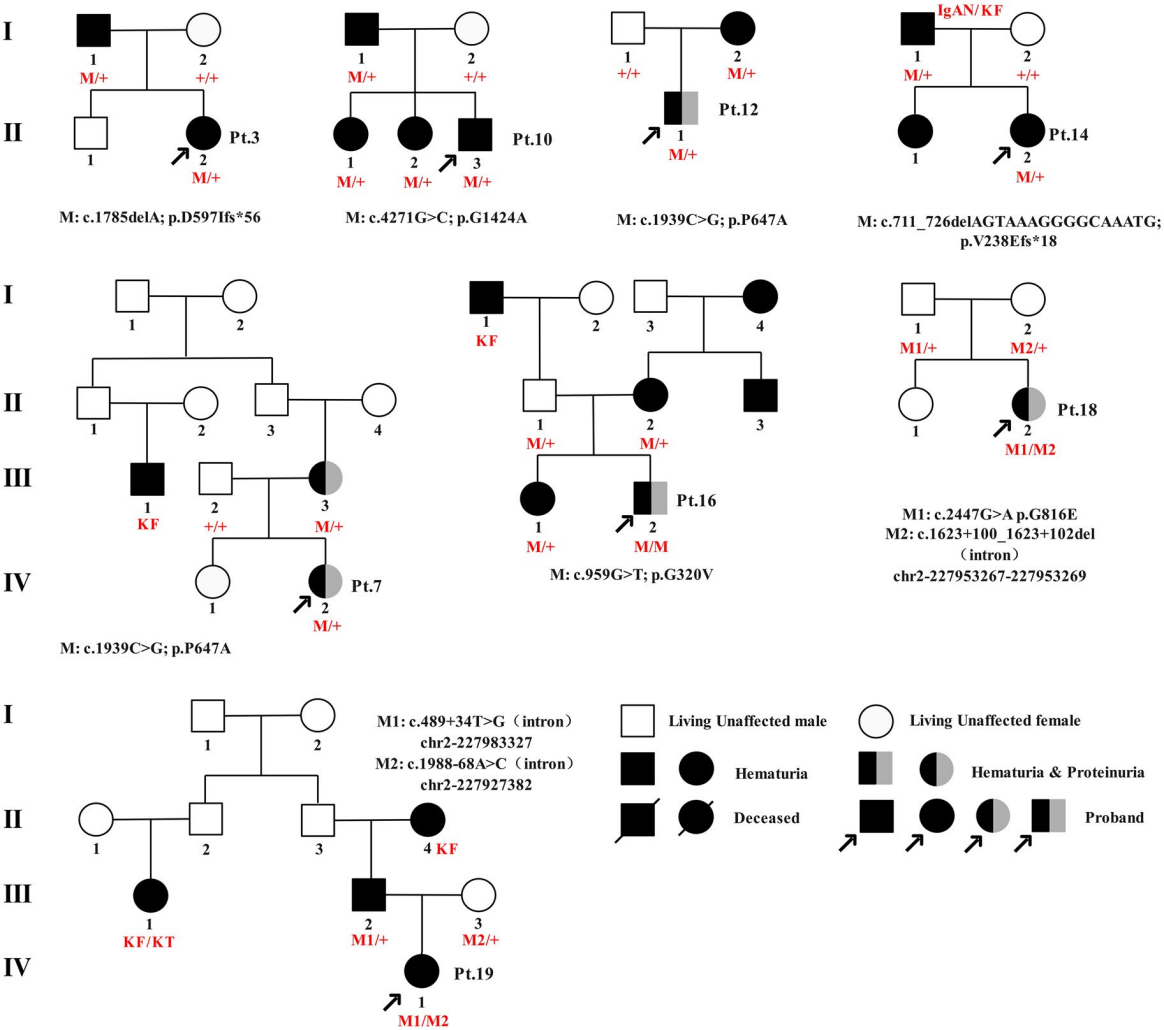
The genealogy of the families with *COL4A4* variants. Black alternating with gray shows patients with hematuria and proteinuria and black indicates patients with isolated hematuria. KF: kidney failure; KT: kidney transplant; IgAN: immunoglobulin A nephropathy.

The classification of AS was closely related to clinical manifestations and pathological findings. Among the 19 children (male/female ratio = 11/8) with *COL4A4* variants, the majority (12/19, 63.2%) were classified as having hematuric Alport kidney disease, a group historically diagnosed as thin basement membrane nephropathy. Three cases (3/19, 15.8%) were classified as Group 3, representing proteinuric or nephrotic Alport kidney disease with focal segmental glomerulosclerosis (FSGS) and four cases (4/19, 21.1%) as Group 2, representing mild to moderate AS. No cases in our study were classified as Group 1, the classic AS group. The median age of first detection of urinary abnormalities was 4.4 years (range: 2.5–6.1 years), and the median age at last follow-up was 9.3 years (range: 7.0–12.7 years) years. Renal phenotypes were primarily characterized by hematuria and proteinuria, with no evidence of progressive kidney failure or extrarenal manifestations typically seen in classic AS. Hematuria was present in all individuals (15 microscopic and 4 gross), while proteinuria was observed in 5 patients (5/19, 26.3%), with two presenting at their first visit and three developing it over time (Table 2). The median age of onset for proteinuria was 7.0 years (2.6–9.6 years). Most patients had a family history of kidney disease or abnormal urine tests, except for Patient-18 (Figure 2 and Supplementary Figure 1). At 1 year and 3 months, Patient-18 presented with eyelid and lower limb edema, foamy urine, and gross hematuria, and was diagnosed with nephrotic syndrome based on laboratory findings of hypoproteinemia and hyperlipidemia. This patient carried distinct *COL4A4* variants inherited from each parent, though no family history of kidney disease was noted.

**Table 2.** Phenotypes of cases with *COL4A4* variants.

Pt. No.	Sex	Genetic variant (cDNA; protein)	Age at onset (y)	Age at last follow-up (y)	Clinical presentation^a^	Biopsy				Family history^c^	Inherited	Group of AS^b^
						Age at renal biopsy(y)	Light microscopy	GBM(α3/α5)	Electron microscopy			
1	M	c.2752G > A (p.G918R)	6.1	6.6	MH	NA	NA	NA	NA	P	AD	4
2	F	c.2317_2318del (p.R773Gfs*14)	4.3	9.3	GH; PRO	4.9	Minor lesion	+/Segmental reduction	Diffusely thin GBM(<180nm)	P	AD	3
3	F	c.1785del (p.D597Ifs*56)	4.8	9.1	MH	NA	NA	NA	NA	P	AD	4
4	M	c.4599T > G (p.Y1533*)	3.2	7.8	MH	4.9	Minor lesion	+/Segmental reduction	Diffusely thin GBM(<180nm)	P	AD	4
5	M	c.290G > A (p.R97H)	2.1	16.3	MH	NA	NA	NA	NA	P	AD	4
6	M	c.4550T > G (p.F1517C)	12.2	14.3	MH	NA	NA	NA	NA	P	AD	2
7	F	c.1939C > G (p.P647A)	2.2	10.6	MH; PRO	6.8	A small number of glomerulosclerosis	+/+	Diffusely thin GBM(<180nm)	P	AD	3
8	M	c.4063G > A (p.G1355R)	3	5	GH	NA	NA	NA	NA	P	AD	4
9	F	c.3506-3_3527del (p.?)	1.5	4.4	GH	NA	NA	NA	NA	P	AD	4
10	M	c.4271G > C (p.G1424A)	4.4	16.3	MH	NA	NA	NA	NA	P	AD	4
11	F	c.4599T > G (p.Y1533*)	5	11.4	MH	NA	NA	NA	NA	P	AD	4
12	M	c.1939C > G (p.P647A)	3.9	7	MH; PRO	NA	NA	NA	NA	P	AD	3
13	M	c.1805G > A (p.G602E)	2.5	13.3	MH	NA	NA	NA	NA	P	AD	4
14	F	c.711_726del (p.V238Efs*18)	6.6	9.6	MH	NA	NA	NA	NA	P	AD	4
15	M	c.4599T > G (p.Y1533*)	5.4	8.9	MH	NA	NA	NA	NA	P	AD	4
16	M	c.959G > T (p.G320V)	4.8	10.8	MH; PRO	NA	NA	NA	NA	P	AD	2
17	M	c.4599T > G (p.Y1533*)	7.5	12.7	MH	NA	NA	NA	NA	P	AD	4
18	F	c.2447G > A (p.G816E)	1.2	3.4	GH; PRO; edema	1.2	FSGS NOS	+/+	GBM approximately 100-200nm thick, segmental wrinkling	N	AD	2
		c.1623 + 100_1623 + 102del (p.?)										
19	F	c.489 + 34T > G (p.?)	6.8	8.4	MH	NA	NA	NA	NA	P	AD	2
		c.1988-68A > C (p.(=))										

NA, not available; y, years; FSGS NOS, focal segmental glomerulosclerosis, non special type; GBM, glomerular basement membrane.

^a^ Clinical presentation: MH, microscopic hematuria; GH, gross hematuria; PRO, proteinuria; The patient-18 also presented with edema of eyelids and lower limbs.

^b^ Group of AS: Group1, severe classic AS; Group2, mild to moderate AS; Group3, proteinuric/nephrotic Alport kidney disease with focal segmental glomerulosclerosis (FSGS).

^c^ Family history: N, negative; P, positive.

Kidney biopsy was instrumental in diagnosing AS. Attending pediatric nephrologists recommended kidney biopsy for children presenting with hematuria along with albuminuria or new-onset albuminuria and four children underwent biopsy with the informed consent of their guardians eventually, and the pathological features are summarized in Table 2. Light microscopy revealed minor lesions, with glomerular sclerosis in Patient-7 and segmental sclerosis in Patient-18. Immunofluorescence showed no evidence of immune complex or complement deposition in any case. Staining for the α3 and α5 collagen chains in the GBM indicated that two cases had normal α3 and α5 expression, while the other two had normal α3 expression but decreased segmental α5 expression. Electron microscopy revealed diffuse GBM thinning (<180 nm) in three cases, while one case exhibited uneven GBM thinning (100–200 nm) with segmental wrinkling.

### 3.3. Genotype-phenotype correlation of COL4A4 variants

**Figure 3.**
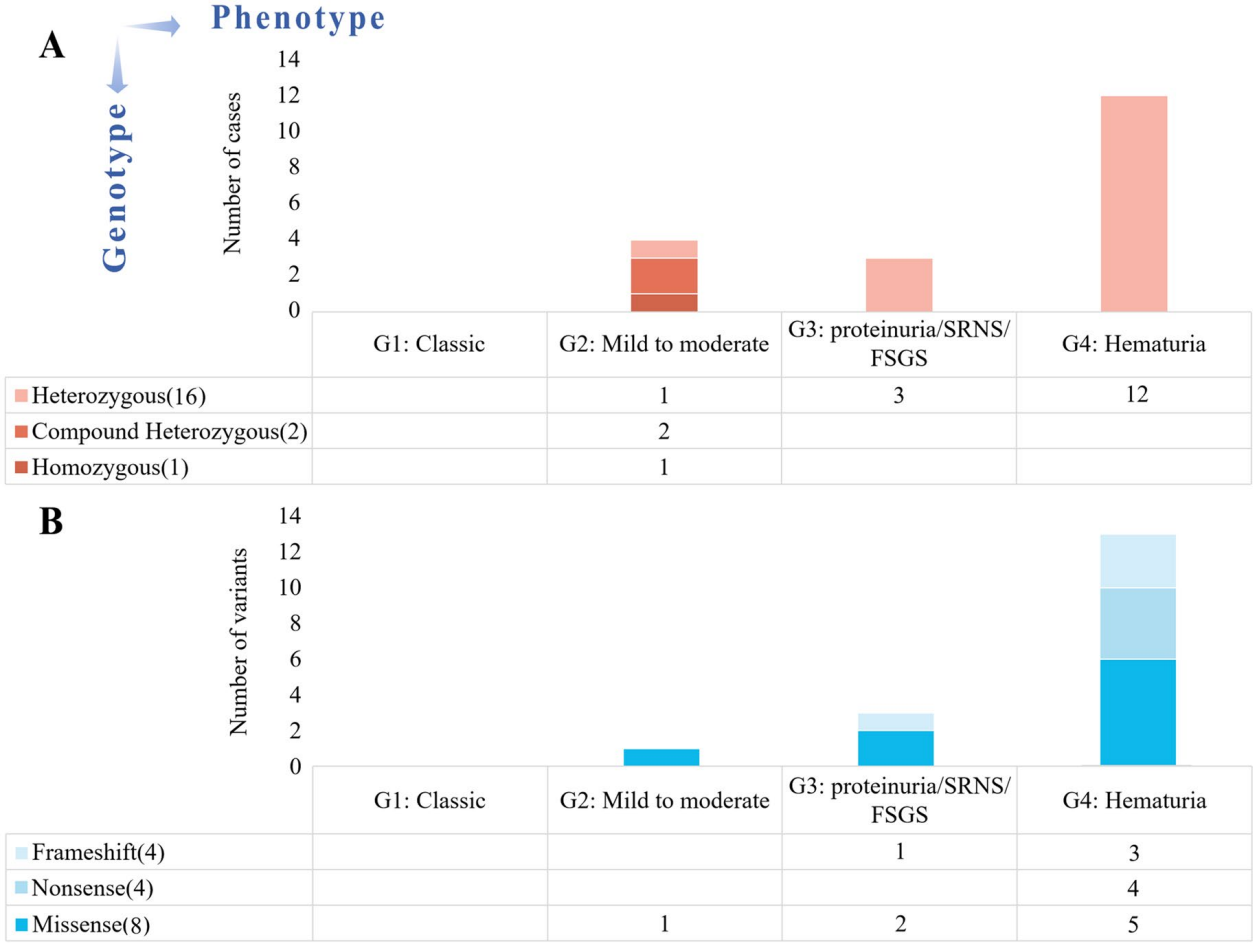
The correlation between genotype and phenotype: (A) the zygosity and (B) the types of detected *COL4A4* variants in heterozygous patients and the classification of AS.

In this study, compound heterozygous and homozygous patients were classified as mild to moderate Alport kidney disease while most heterozygous patients were classified as proteinuric and hematuric Alport kidney disease (Figure 3A). In heterozygous patients, frameshift and missense variants in *COL4A4* were associated with varying degrees of Alport kidney disease, and nonsense variants resulted in isolated hematuria (Figure 3B). Group 3 and Group 4 contained the most diverse variant classifications. In our cohort, no patients with *COL4A4* variants had been classified as classic AS group. No statistically significant difference was observed in the age of onset (first detection of urinary abnormalities) between truncating and non-truncating variants in heterozygous patients (*p* = 0.382).

## 4. Discussion

The six highly homologous chains of type IV collagen (α1–α6), encoded by *COL4A1*–*COL4A6*, form three distinct heterotrimers: α1α1α2(IV), α3α4α5(IV), and α5α5α6(IV) [13]. The α1α1α2(IV) heterotrimer is predominant in all basement membranes during embryogenesis, while the α3α4α5(IV) heterotrimer becomes the dominant component in mature glomerular basement membranes (GBMs) and in the basement membranes of the cochlea and ocular lens [14]. Consequently, variants in type IV collagen genes can impair basement membranes, leading to the thinning, splitting, or layering of GBMs, which are hallmark features of Alport syndrome (AS) [15]. In a cohort of Chinese children with AS, most presented with glomerular hematuria and varying degrees of proteinuria, with minimal extrarenal involvement [5]. In one single-center study of children with nonselective hematuria, 11.9% (5/42) were found to carry *COL4A4* variants [15].

In this study, we identified 10 distinct novel *COL4A4* variants (10/17). Of these, one variant (Patient-14) was classified as pathogenic, while three (Patients 3, 10, and 16) were deemed likely pathogenic according to ACMG guidelines. The frameshift variants p.V238Efs18 and p.D597Ifs56 were caused by the deletion of 16 amino acids at the Intron37-Exon38 junction and the loss of an adenine at position 1785, respectively. Species conservation analysis suggested that the missense variants p.G1424A (Patient-10) and p.G320V (Patient-16), located in the triple-helical region, likely destabilize the collagen structure due to their effects on highly conserved amino acids. For variants of uncertain significance, different functional modeling prediction tools such as SpliceAI were used to conduct pathogenicity assessment (Table 1 and Supplementary Table 1). The missense variant p.P647A was predicted to be damaging by SIFT and PolyPhen-2, warranting caution when interpreting its pathogenicity. For c.489 + 34T > G, SpliceAI indicated the potential activation of a cryptic donor site (DS_DG = 0.90), which may result in abnormal splicing events. For c.1623 + 100_1623 + 102del, HSF Pro predicted the activation of a cryptic acceptor site and the potential alteration of splicing. The comparison of the sites and clinical manifestations between the new variants and the reported pathogenic *COL4A4* variants was shown in Supplementary Table 3.

We identified two compound heterozygous variants and one homozygous variant. In the family of Patient-18, only the proband, diagnosed with steroid-resistant nephrotic syndrome at age 1, exhibited renal pathology consistent with focal segmental glomerulosclerosis (FSGS). This patient carried a paternal missense variant (c.2447G > A/p.G816E), which had been previously reported in association with steroid-resistant nephrotic syndrome [16], yet her father, who carried the same variant, was asymptomatic. This finding aligns with the ACMG classification of unknown clinical significance. Another unreported intronic variants (c.1623 + 100_1623 + 102del), inherited from her asymptomatic mother, was also classified as VUS. In another compound heterozygous patient (Patient-19) with two intronic variants, a family history of kidney failure was noted, though genetic testing was unavailable for affected relatives (Figure 2). In Patient-16’s family, carriers of a homozygous variant displayed a mild renal phenotype or were asymptomatic, possibly indicating potential influences of gender and environmental factors on gene penetrance.

Interestingly, the same genotype led to different phenotypes. For instance, Patient-7, carrying the unreported p.P647A variant, experienced an earlier onset of symptoms compared to Patient-12, despite both having family histories of kidney disease. Patient-7’s biopsy revealed FSGS, and she has been treated with ACE inhibitors (ACEI) since age 7. In contrast, Patient-12, who started ACEI treatment at age 4, has shown normal urinalysis and kidney function two years after discontinuing the medication. Although classified as VUS, the family history and clinical findings suggest that the p.P647A variant warrants careful consideration. In our cohort, all patients carrying the pathogenic nonsense variant (c.4599T > G/p.Y1533*) which had been reported in a homozygote, presented with hematuria but not extrarenal manifestations [17], suggesting that this variant may be associated with a milder phenotype when heterozygous.

To our knowledge, this is the first study to apply the revised Alport syndrome classification in a cohort of Chinese pediatric patients with *COL4A4* variants, expanding the spectrum of the *COL4A4* variants and provides insight into genotype-phenotype correlations in pediatric patients. To reinforce the translational and scientific value of our work, all new *COL4A4* variants (*n* = 10) verified by Sanger will be uploaded to ClinVar in accordance with the HGVS nomenclature specification. The curated set of structured genotype-phenotype pairs (Table 2) derived from this cohort has the potential to train AI models and integrate into the phenotype-prediction pipelines in nephrogenetics.

At present, international databases such as ClinVar and ACMG guidelines are mainly based on European and American data, and the public database like gnomAD is underrepresented for non-Western populations, which leads to deviations in the interpretation of variant pathogenicity in non-Western ­populations. Our work is conducive to establishing a *COL4A4* variant spectrum database in the Chinese population, which can significantly reduce missed diagnoses caused by variant misreading and make contributions to precision medicine. The findings may also inform genetic counseling and variant interpretation practices. The identification of novel *COL4A4* variants with associated phenotypic profiles can support more precise genetic counseling in children presenting with hematuria, helping to distinguish between TBMN and early-stage Alport syndrome. Besides, longitudinal follow-up provides time-resolved phenotypic evidence to empower future variant-reclassification efforts, particularly for variants of uncertain significance, by contributing real-world evidence of phenotypic expression over time. Additionally, the study provides population-specific allele frequency data which is valuable for precision nephrology and adds to the understanding of disease presentation in East Asian populations, underscoring the need for population-specific variant databases in nephrology to improve diagnostic accuracy and reduce health disparities in genomic medicine. Finally, our findings support the utility of early genetic testing in children with hematuria to guide surveillance and family counseling.

However, several limitations must be considered when interpreting the results. First, owing to the sample size (*n* = 19) drawn exclusively from one center and limited to pediatric patients only with *COL4A4* variants, the generalizability of our results is curtailed. Second, the limited sample leads to the potential for underpowered statistical comparisons. Taken together, the findings must be interpreted cautiously until replicated in larger, multi-center cohorts. Future studies are needed to further elucidate the clinical course of Alport kidney disease with *COL4A4* variants: (i) prospective longitudinal cohorts validating genotype-phenotype correlations, (ii) functional studies of novel *COL4A4* variants (splicing, protein modeling), (iii) AI-driven phenotype prediction pipelines trained on structured variant-phenotype datasets, (iv) population-specific variant databases in East Asia to reduce misclassification bias.

## Supplementary Material

Supplementary Table1.xlsx

Supplementary Table3.xlsx

Supplementary Figure1.tif

Supplementary Table2.xlsx

## Data Availability

The data in this study are available on reasonable request from the corresponding author.
