# Supplementary figures and images for: Expanding the COL4A4 variant spectrum: genotype-phenotype correlation in 19 Chinese children using updated Alport kidney disease classification
Source: Ren Fail. 2025 Oct 14;47(1):2570072. doi: 10.1080/0886022X.2025.2570072 (PMC12523472; doi:10.1080/0886022X.2025.2570072)

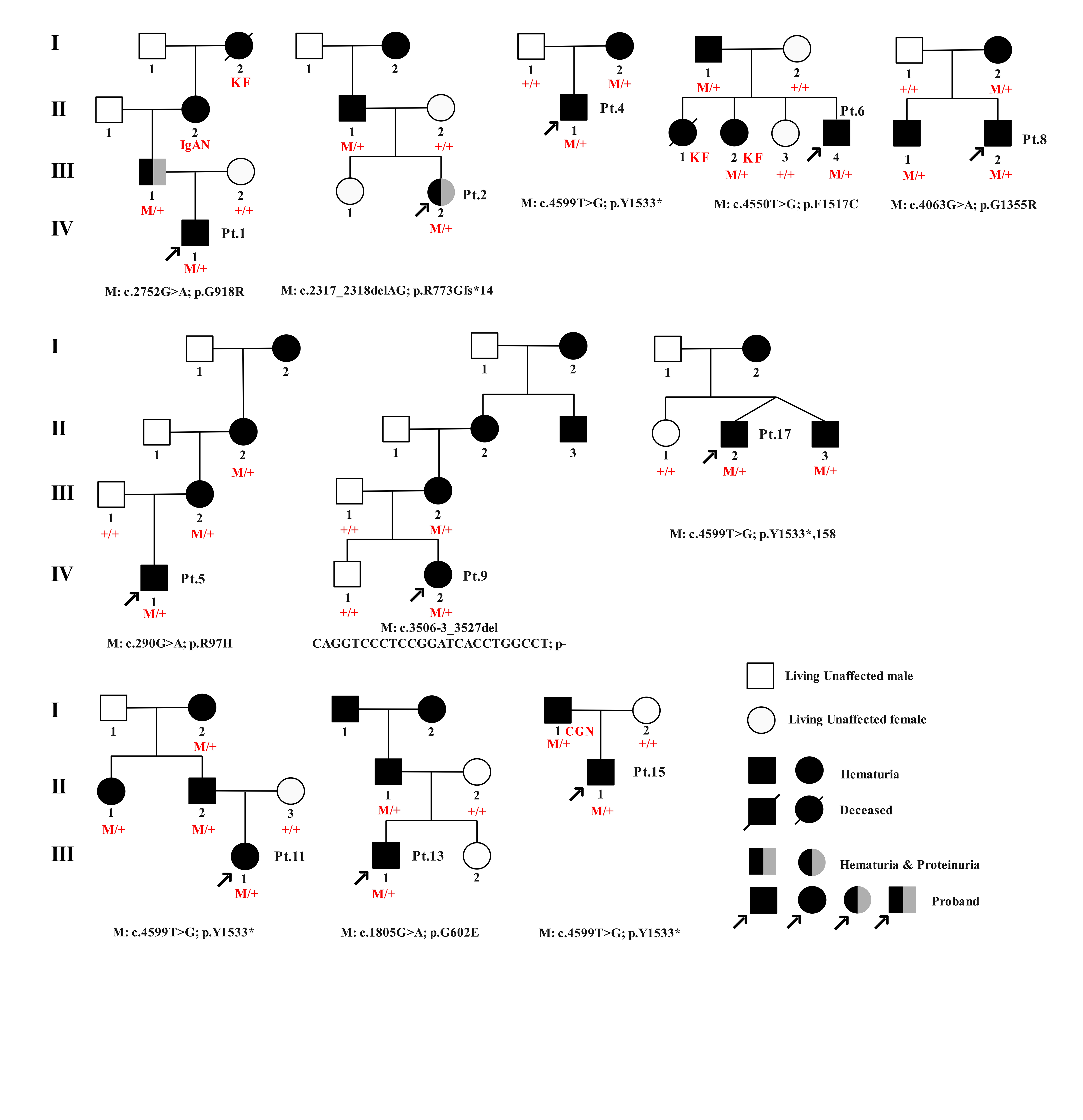

Supplement: Supplementary Figure1.tif [file IRNF_A_2570072_SM8036.tif]
